# Nanoparticles for Targeted Brain Drug Delivery: What Do We Know?

**DOI:** 10.3390/ijms222111654

**Published:** 2021-10-28

**Authors:** Rúben G. R. Pinheiro, Ana Joyce Coutinho, Marina Pinheiro, Ana Rute Neves

**Affiliations:** 1 LAQV, REQUIMTE, Departamento de Ciências Químicas, Faculdade de Farmácia, Universidade do Porto, Rua de Jorge Viterbo Ferreira 228, 4050-313 Porto, Portugal; 2 CQM—Centro de Química da Madeira, Campus da Penteada, Universidade da Madeira, 9020-105 Funchal, Portugal

**Keywords:** Alzheimer’s disease, drug delivery, drug targeting, neurodegenerative diseases, Parkinson’s disease

## Abstract

The blood–brain barrier (BBB) is a barrier that separates the blood from the brain tissue and possesses unique characteristics that make the delivery of drugs to the brain a great challenge. To achieve this purpose, it is necessary to design strategies to allow BBB passage, in order to reach the brain and target the desired anatomic region. The use of nanomedicine has great potential to overcome this problem, since one can modify nanoparticles with strategic molecules that can interact with the BBB and induce uptake through the brain endothelial cells and consequently reach the brain tissue. This review addresses the potential of nanomedicines to treat neurological diseases by using nanoparticles specially developed to cross the BBB.

## 1. Introduction

The blood–brain barrier (BBB) is a physical barrier, with unique properties, that is present in the majority of blood vessels irrigating the brain. It tightly regulates the access of ions, molecules, and cells coming from the blood to the central nervous system (CNS) [1]. The unparalleled architecture of the BBB, characterized by a layer of continuous nonfenestrated endothelial cells surrounded by smooth muscle cells, pericytes, and projections of astrocytes, is essential for its function [1]. In steady state conditions, the BBB is critical to protect brain from harmful agents, but the other side of the coin is that its high selectivity makes access difficult for the of drugs used in the treatment of many neuronal disorders. In that sense, the challenge of crossing and transposing the BBB is crucial for an effective and successful therapy. Nanotechnology is an essential tool when developing new systems for the efficient delivery of potential therapeutic and diagnostic compounds to specific areas of the brain, since they reduce the adverse side effects associated with the non-specific distribution of drugs, increase drug concentration at the desired site of action and, consequently, improve the therapeutic effectiveness [2,3,4,5,6]. Therefore, nanoparticles (NPs) have gained increasing attention in the field of medicine, being considered a promising tool for drug development [2]. Various kinds of NPs, namely, liposomes, lipid nanoparticles, polymeric nanoparticles, dendrimers, cyclodextrins, silica nanoparticles, magnetic nanoparticles, gold nanoparticles, quantum dots, and carbon nanotubes, have been described as appealing candidates for increasing the penetration of drugs through BBB. The suitability of nanodelivery systems for brain delivery depends on properties such as nanometric size, surface charge, morphology, and, especially, the molecular recognition and interaction between a specific ligand conjugated on the nanoparticle surface and the molecule overexpressed on the brain target place (active targeting) [2]. The intrinsic characterization parameters of NPs, including the determination of size, zeta potential, and encapsulation efficiency (EE) of the NPs, play a pivot role in their biological effects and ultimately in their efficacy and safety [2]. The nanoscale is very important because the order of magnitude enables the interaction with cells, leading to a cell response. Therefore, the size and composition of nanosystems can be controlled and adjusted in order to create particles capable of passing through the BBB [3]. Most studies report a size ranging from 100 to 300 nm as the most appropriate for NP transport of drugs across the BBB [4]. Additionally, one should guarantee that they are biocompatible and biodegradable, so that one can minimize toxicity in vivo [5]. At the same time, nanosystems appear to be important for reducing the adverse side effects associated with the non-specific distribution of drugs, increasing drug concentration at the desired site of action in the brain, and, consequently, improving the therapeutic effectiveness [6].

## 2. Active Targeted Brain Delivery

**Figure 1:**
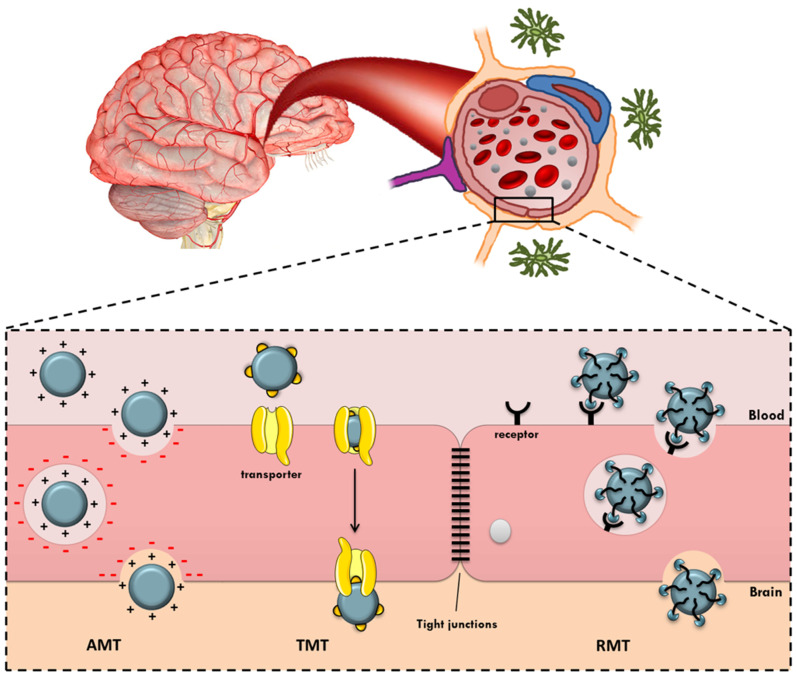
Schematic representation of the different mechanisms of nanoparticles for crossing the blood–brain barrier (BBB). AMT, adsorptive-mediated transcytosis; TMT, transporter-mediated transcytosis; RMT, receptor-mediated transcytosis.

Active targeting is particularly important when designing solutions to achieve this goal, since this strategy allows directing the nanoparticles to the desired place and, consequently, transporting and delivering drugs to the site of action, the brain. In fact, these nanosystems display a great surface area to volume ratio, which enables the nanoparticles to be highly chemically reactive, allowing surface modification with molecules that may be recognized by the receptors/transporters overexpressed in the BBB and cell-specific receptors in the brain tissue [7,8]. There are, essentially, three different strategies for achieving this purpose (Figure 1): adsorptive-mediated transcytosis, transporter-mediated transcytosis, and receptor-mediated transcytosis [9,10]. Once in the brain, it is necessary that nanoparticles can reach the desired target, such as brain tumor cells, neurons, or even the fibrils associated with many neurological diseases. Different examples of nano-drug delivery systems described in the literature for brain delivery are listed in Table 1.

### 2.1. Adsorptive-Mediated Transcytosis

Adsorptive-mediated transcytosis (AMT) provides a route for the brain delivery of nanoparticles across the BBB. The endothelial cells of BBB constitute a phospholipid rich membrane covered by a glycocalyx composed of heparan sulfate proteoglycans (HSPGs), namely glypican and syndecan [11]. Moreover, there are many carboxyl groups of sialoglycoproteins and sialoglycolipids on this side of the BBB [12]. Together, these make the luminal side of the BBB highly negatively charged. Therefore, AMT can be promoted using the electrostatic interactions between the negative moieties exposed at the luminal surface of cerebral endothelial cells and the cationic groups of ligands conjugated on the nanoparticles surface [13]. Several studies have demonstrated that clathrin-coated pits or caveolae are involved in AMT, since after charge–charge interactions, the nanoparticles will then cross brain endothelial cells through the formation of membrane-invaginated vesicles [14,15]. In fact, the brain capillary endothelium is particularly enriched in clathrin-coated pits and caveolae compared to the peripheral endothelia. However, it is important to mention that the clathrin-coated pits are more abundant than the caveolae, indicating that transcytosis occurs mainly by clathrin-mediated mechanisms [16]. Basically, clathrin-coated pits along the luminal surface of endothelial cells are negatively charged and thus are capable of binding positively charged nanoparticles. Therefore, this strategy takes advantage of electrostatic interactions between nanoparticles and their target. Nanoparticles can be functionalized with positively charged molecules, in order to induce BBB adsorption and transcytosis of the nanocarrier into the brain. However, by itself, AMT does not ensure cell-specific targeting, since positively charged molecules can be rapidly and indiscriminately adsorbed by all negatively charged cell membranes and, thus, penetrate a variety of different cells. Nevertheless, several works reported the use of AMT as a strategy for brain delivery.

#### 2.1.1. Lectin

Lectins are proteins or glycoproteins of nonimmunological origin that specifically recognize sugar molecules and, therefore, are capable of binding glycosylated membrane components. These proteins are positively charged and, hence, theoretically they might establish electrostatic interactions and adsorb to the BBB, increasing brain uptake. Although very promising, this strategy is more common for nose to brain delivery, which bypasses the BBB. Nonetheless, there are many works using lectin mediated-transport for brain targeting [17,18,19,20,21]. In a primary work, Gao et al. synthesized polymeric nanoparticles composed of PLA and functionalized with wheat germ agglutinin and tested the brain uptake in intranasally administered rats [17]. The results showed a negligible nasal toxicity of these nanocarriers and, at the same time, an increase of brain uptake [17]. This system could be very promising for treating neurodegenerative diseases, because it can increase drug concentrations in the brain and drastically reduce the side-effects. Zhang et al. conducted a work using PLA nanoparticles coated with solanum tuberosum lectin to encapsulate basic fibroblast growth factor [18]. These nanoparticles were nasally administered in AD rats, and an increase in spatial learning and memory by the Mirror water maze task was reported [18]. Moreover, in a study with hemiparkinsonian rat models, PLGA nanoparticles functionalized with odorranalectin (less immunogenic) and loaded with urocortin peptide were able to reduce rat rotational behavior, demonstrating a neuroprotection capacity [20]. This could be an attractive approach, not only in neurodegenerative diseases, but also in other brain dysfunctions, such as psychiatric problems, because the common medications are associated with a lot of side-effects. Concerning schizophrenia, Piazza et al. developed polymeric nanoparticles composed of PLGA and lectin to encapsulate haloperidol, which were administered intranasally in rats [19]. The results demonstrated an increase of haloperidol in the brain of rats compared to the nanoparticles without a lectin coating [19]. Collectively, these studies show that once in the brain, lectin conjugated nanoparticles are promising for the treatment of neurological diseases, although it remains elusive if they are truly capable of increasing BBB passage.

#### 2.1.2. Cardiolipin

Cardiolipin is an important component of the inner mitochondrial membrane, and it is important for numerous enzyme activities involved in mitochondrial energy metabolism. This molecule is positively charged and, thus, can also induce adsorption to the BBB. This type of approach has been used in some works [22,23,24,25]. Obviously, neurodegenerative diseases emerge as an immediate candidate, and many works have attempted to deliver drugs to the brain to treat the neuronal death associated with these kinds of diseases. Bereczki et al. used liposomes composed of sphingomyelin, cholesterol, and cardiolipin in mouse neuroblastoma cell lines [22]. The results were very promising, since liposomes were capable of rescuing cell viability and, at the same, a reduction in Tau phosphorylation was observed [22]. Moreover, in order to test the hypothesis that alterations in the equilibrium of Aβ levels between plasma and the brain can have a beneficial effect on the treatment of AD, Ordóñez-Gutiérrez and colleagues conducted a study with APP/PS1 transgenic mice (AD model), repeatedly injecting intraperitoneally liposomes composed of sphingomyelin, cholesterol, and cardiolipin [23]. Although the results showed a significant decrease in Aβ levels in the plasma, the levels in the brain were only slightly affected. The same result, in terms of Aβ levels in the brain, was obtained by Taylor et al., using a similar liposome formulation of sphingomyelin, cholesterol, and cardiolipin [24]. However, a reduction of Tau phosphorylation was confirmed, indicating that the modulation of Aβ in plasma may be important to treat AD [23]. Interestingly, although liposomes with cardiolipin cannot by themselves decrease Aβ fibrils in the brain, they reveal a high affinity for these fibrils, as reported by Gobbi et al., using surface plasmon resonance (SPR) experiments [25]. This could be particularly interesting for the generation of new vehicles for imaging and new therapeutic agents.

#### 2.1.3. Heparin

Heparin is a polyanionic polysaccharide, belonging to the glycosaminoglycans (GAGs) family. This polymer has been used for cancer targeting, anti-coagulation, tissue engineering, and drug delivery applications. Heparin’s capacity to compete with Aβ peptides in binding to proteoglycans is not yet well understood. However, this property could be very useful in AD therapy. Wang et al. have studied this property using magnetic nanoparticles (SPION) coated with heparin and showed concurrent binding with Aβ peptides by a variety of techniques, including enzyme-linked immunosorbent assay, gel electrophoresis, and thioflavin T assay [26]. Moreover, they observed that these nanoparticles were capable of protecting neuronal cells from Aβ toxicity, demonstrating that this nanosystem could be very promising [26].

#### 2.1.4. Cell Penetrating Peptides

Cell penetrating peptides (CPP) are small peptides that can be translocated across cellular membranes and gain access to the intracellular space [27]. All these peptides possess several positive charges, as well as other shared properties, such as their amphipathic behavior and ability to interact with lipid membranes [28]. According to the literature, the internalization of CPP does not depend on a specific binding to a receptor [29,30,31]. Although the exact mechanism has not yet been precisely described, it seems that electrostatic interactions between the lipid membranes or other components (glycoproteins and glycosaminoglycans present in extracellular matrix) and CPP induce the endocytosis and, consequently, the internalization [32,33,34]. This is a subtle strategy for overcoming the problem of crossing the BBB and, therefore, there are many works reported in the literature using this approach [35,36,37]. The efficiency of these peptides for brain delivery was tested in a study conducted by Xia et al., using penetrating-functionalized PEG–PLA nanoparticles in Sprague-Dawley rats [36]. These nanocarriers were able to increase brain uptake and, at the same time, reduced the accumulation in non-targeted organs [36]. This CPP specific targeting was also confirmed in a study conducted by Santra et al., with quantum dots functionalized with TAT using intra-arterially injected rats [37]. This ability for targeting brain is also very useful in the cancer field, because it is crucial for solving the problem related to the side-effects associated with therapeutic agents. Simultaneously, it is also important to find new approaches to increase our efficacy in cancer treatment. Having this in mind, Kanazawa et al. synthesized polymeric micelles composed of poly(ε-caprolactone) coated with TAT and loaded with siRNA for Raf-1/anticancer drug camptothecin, even though, in this particular case, the intranasal administration in a rat model of malignant glioma did not require BBB passage [35]. The silencing of Raf-1 through siRNA impacted cell proliferation and apoptosis, increasing survival and inhibiting tumor growth [35]. Moreover, the viability of normal neuronal cells was preserved, indicating a specific targeting of cancer cells and making this a very promising strategy in the brain cancer field [35]. Taking into consideration that the latter strategy has shown great efficacy in glioma treatment, it is conceivable that others routes requiring BBB passage are feasible, since the use of CPP for this purpose has already been described, as cited above.

### 2.2. Transporter-Mediated Transcytosis

An alternative strategy for the brain delivery of drugs is the use of BBB-specific transporters, for efficient supply of nutrients of low molecular weight from the bloodstream to the CNS. Twenty different transporters are well known in the BBB [38,39]. In that sense, transporter-mediated transcytosis (TMT) could be an important approach for the design of nanocarriers for brain delivery. In fact, it is possible to synthesize nanoparticles with molecules conjugated on their surface that are well recognized by the transporters overexpressed in brain endothelial cells. However, this approach is not widely used, because it can interfere with the normal uptake of nutrients [10]. Nevertheless, the most common strategies are the use of glucose, glutathione, and amino acids transporters.

#### 2.2.1. Glucose Transporter 1

Glucose transporter 1 (GLUT1) is a transmembrane protein that belongs to the GLUT family, with 14 different members known in humans, to date [40]. This transporter is expressed in the BBB, in order to mediate the passage of metabolic substrates, such as sugars [39,41]. Furthermore, GLUT1 seems to be overexpressed in brain cancer cells, which makes it an attractive candidate target for glioma treatment [42,43,44]. Some authors have used this transporter to facilitate permeation through the BBB and deliver drugs inside the brain [45,46]. Singh et al. tested the efficacy of lipid nanoparticles modified with MAN, which is an analogue of mannose with high affinity for GLUT transporters. With this approach the authors were interested in delivering docetaxel, a chemotherapy agent, to the brain [45]. The results demonstrated an enhancement in brain uptake, intravenously injected mice, demonstrating excellent potential for brain cancer drug delivery [45]. In a more in-depth study, cationic HSA nanoparticles were modified with ethylenediamine and mannose and loaded with doxorubicin [46]. The in vitro results showed an enhancement in BBB uptake, as well as in U87MG glioblastoma cells [46]. The in vivo results using intravenously injected glioma-bearing mice were also in agreement with the previous evidence, since the mice treated with mannose-coated HSA nanoparticles displayed a reduction in tumor size, when compared to animals treated with uncoated HSA nanoparticles [46].

#### 2.2.2. Glutathione Transporter

Glutathione (GSH) is a hydrophilic endogenous tripeptide and well-known antioxidant. GSH has been studied in recent years as a potential candidate to facilitate the transporter-mediated transcytosis of nanocarriers, by interacting with a family of ATP-binding cassete (ABC) transporters, named multidrug resistance proteins (MRP), on BBB cells, such Mrp1, Mrp2, and Mrp5 [47]. Therefore, some works have demonstrated that GSH-coupling nanoparticles represent a feasible and promising approach for the delivery of drugs to the brain [48,49,50]. Maussang et al. used liposomes modified with GSH and increased the brain uptake of ribavirin, which was used as a drug model, in intravenously injected rats [48]. Furthermore, the uptake mechanism was studied using human, bovine, and porcine cerebral microvascular endothelial cells, showing a temperature-, time-, and dose-dependent endocytosis, mediated via clathrin coated pits [48]. The same enhanced brain uptake was obtained with GSH-coated PLGA nanoparticles loaded with paclitaxel in intranasally injected rats; albeit, in this example using a direct-transport nose to brain bypass BBB passage [49]. Concerning gene delivery, Englert et al. optimized a cationic polymeric nanocarrier composed of poly(ethyleneimine), and modified with GSH, and tested this system in a hCMEC/D3 endothelial cell layer and mimicking the BBB within microfluid-perfused biochips [50]. The cationic nanocarriers were able to cross the monolayer and release the DNA plasmids [50]. Hence, these studies demonstrate the capacity of GSH-modified nanoparticles to target the brain.

#### 2.2.3. Amino Acids Transporters

Amino acids transporters participate in the uptake of amino acids by cells and can be classified in various systems, according to their substrate selectivity. For instance, L-type amino acid transporter 1 (LAT1) is overexpressed in many cancer cells and is responsible for transporting large neutral amino acids [51]. In particular, LAT1 is highly expressed in the BBB and in brain cancer cells [52,53]. Therefore, this transporter seems to be an interesting target for mediating BBB passage and, consequently, targeting brain cancer cells at the same time. In this context, some authors have tried to develop this approach for brain tumor delivery [54,55]. This dual-targeting strategy was used as a proof of concept by Li et al. [54]. Fluorescent dyes (DIR and coumarin) were encapsulated inside liposomes modified with glutamate, and the results revealed a higher uptake by C6 glioma cells in vitro, compared to unmodified liposomes [54]. Additionally, intravenously injected mice revealed a DIR accumulation in the brain, which attested to the capacity of this transporter to allow BBB passage [54]. In the same line, Kharya et al. examined the capacity of solid lipid nanoparticles modified with phenylalanine, and loaded with doxorubicin, for brain and tumor targeting [55]. The results illustrated an enhancement of C6 glioma cell uptake and an increase of doxorubicin brain accumulation in intravenously injected mice. Therefore, such amino acids transporters can effectively facilitate the transport of nanoparticles across the BBB and increase access to the brain tumor microenvironment [55].

### 2.3. Receptor-Mediated Transcytosis

Another, alternative, way of reaching the brain tissue is taking advantage of receptors overexpressed in the BBB; the so-called receptor-mediated transcytosis (RMT). This mechanism induces endocytosis through clathrin-coated pits or caveolae, resembling the process in AMT [41]. After internalization of nanoparticles, they can follow different pathways inside cells, depending on their size, charge, composition, and ligand-conjugation [41]. The most common receptors used to mediate RMT through the BBB are transferrin, lactoferrin, low density lipoprotein, and nicotinic acetylcholine receptors, which will be discussed below. Additionally, the expression of insulin receptor and insulin-like growth factor receptor on the luminal membrane of brain capillary endothelial cells might also be an interesting approach [56]. This strategy was attempted in rhesus monkeys, where pegylated immune liposomes were conjugated with monoclonal antibody against human insulin receptor (HIR) and loaded with plasmids encoding either luciferase or β-galactosidase. Indeed, the results confirmed the widespread expression of β-galactosidase in the primate brain. Nevertheless, the risk of affecting the natural insulin balance has impaired the widespread use of these receptors in the nanotechnology field [57].

Having this in mind, nanoparticles can be modified with specific ligands of receptors and, hence, can be taken up by brain endothelial cells. Once in the brain, nanoparticles have to reach the right cell target, and active targeting can also be designed for this purpose. Nicotinic acetylcholine receptors can be used for targeting neuronal cells, while αvβ3 integrin and CD13/APN receptors are well-known examples of receptors overexpressed in the brain tumor microenvironment, allowing the development of novel therapies that specifically target the cancer cells in the brain tissue [58,59].

#### 2.3.1. Transferrin Receptor

Transferrin receptor (TfR) is a membrane protein composed of two identical glycosylated subunits, which dimerize and bind to the circulating transferrin [60]. This binding induces endocytosis via clathrin-coated pits and ensures the iron uptake to the brain, because this metal serves as a co-factor for many enzymes and participates in many metabolic processes, such as ATP synthesis, as part of the iron–sulfur cluster and heme group of mitochondrial respiratory chain complexes [61]. TfR is expressed in the endothelial cells of the brain, but the endothelial cells present in different tissues cannot express this receptor, making it a very interesting approach for mediating BBB passage and delivering drugs to the brain [62]. Taking advantage of these receptors, there are plenty of examples in the literature of nanoparticles functionalized with transferrin in order to increase the brain uptake of therapeutic drugs [63,64,65,66,67,68,69]. Intending to test the potential of this approach, Kuo et al. used a transwell chamber with a brain-microvascular endothelial cell (HBMEC) monolayer and examined the capacity of the cationic lipid nanoparticles, composed by stearic acid, compritol, and transferrin, to pass through this in vitro model of the BBB [63]. The results showed the uptake and transcytosis of the nanocarrier through the HBMEC, observed by cytoplasmic fluorescence [63]. Consistently, the uptake by brain endothelial cells was also verified with PLGA nanoparticles functionalized with transferrin in an in vitro study conducted by Chang et al. [64]. Transposing this approach into an in vivo experimental model, Li and colleagues confirmed this hypothesis using carbon dots functionalized with transferrin in an intravenous-injected zebrafish model, in contrast to what happened with carbon dots alone [65]. Therefore, the concept of transferrin as a mediator of transportation across the BBB by a RMT process could allow the design of nanocarriers loaded with therapeutic drugs, which will be delivered in the brain. In accordance with this, albumin nanoparticles functionalized with transferrin and loaded with loperamide were used by Ulbrich et al. in intravenously injected ICR (CD-1) mice [66]. This drug has anti-nociceptive effects and cannot naturally cross the BBB. Nevertheless, using the tail-flick test, which consists of a pain response test, it was possible to confirm the brain delivery capacity of this nanocarrier system [66]. Moreover, liposomes conjugated with transferrin and loaded with α-Mangostin enhanced targeted delivery in the brain of intravenously injected rats, demonstrating the ability to cross the BBB [67]. Obviously, this strategy is also very appealing in the brain cancer field, because it is necessary to find better approaches to deliver drugs precisely to the tumor site. Cui et al. used a complex strategy that conjugates magnetic, polymeric, and mesoporous silica nanoparticles. Taking advantage of the loading capacity and tunable pore size of silica nanoparticles and applying an external magnetic field, they were able to deliver, in a tissue-specific manner, doxorubicin and paclitaxel in a U-87 MG-luc2 xenograft of BALB/c nude mice, an experimental animal model for brain cancer [68]. The results suggested that nanoparticles loaded with doxorubicin had the strongest inhibition of tumor growth, suggesting that this strategy holds great potential for future applications [68]. Another appealing strategy for treating several diseases is the artificial manipulation of cell transcriptome, as idealized by gene therapy. Somani et al. developed 3-diaminobutyric polypropylenimine-transferrin dendrimers loaded with a plasmid for β-galactosidase [69]. This nanosystem was tested in intravenously injected mice, duplicating β-galactosidase expression in the brain compared to the unmodified dendriplex, and indicating that this nanosystem was capable of passing the BBB and modulating neuronal expression; making it very promising for gene therapy [69].

In an alternative to transferrin, nanoparticles can be modified with monoclonal antibodies (OX26 or R17217) against TfR [70]. These monoclonal antibodies have a different binding site on TfR and, therefore, do not interfere with transferrin binding at physiological conditions, which represents a tremendous advantage, because normally the transferrin binding site is saturated [62]. Indeed, some authors adopted this approach in order to avoid the saturation of TfR [66,71,72,73,74]. For example, Ulbrich and coauthors used two coating strategies (transferrin and OX26 mAb) in human serum albumin nanoparticles loaded with loperamide. A comparison between the two strategies showed that monoclonal antibody functionalization had the same efficiency, regarding its capacity to overcome the problem imposed by the BBB, with the advantage of not saturating TfR [66]. Additionally, the same brain-target efficacy of OX26 mAb functionalization was obtained in lipid nanocapsules in intravenously-injected rats [71]. This strategy could be also very advantageous in neurodegenerative disease therapy, where monomer aggregation induces oxidative stress and inflammation, leading to neuronal cell death. In a recent work, PLGA nanoparticles were coated with OX26 mAb and anti-Aβ mAb. Loureiro et al. made a proof of concept for AD using this nanosystem loaded with iAβ5, which inhibits the interaction between amyloid beta peptides [72]. Porcine brain capillary endothelial cells were used as an in vitro model of BBB, and nanocarriers increased the endothelial cells uptake, making this a very promising approach to treat AD [72]. Regarding PD, liposomes conjugated with OX26 mAb and loaded with a plasmid encoding tyrosine hydroxylase were synthesized and administered in PD rat models [73]. This rat model had a 90% reduction in striatal tyrosine hydroxylase enzyme activity due to a neurotoxin administration, but the developed nanocarriers were capable of entering the CNS, rescuing gene expression, and reverting motor impairment, being an excellent approach for brain delivery and gene therapy [73].

#### 2.3.2. Lactoferrin Receptor

Despite the similarities between lactoferrin and transferrin, they differ in their receptor binding properties. This feature is due to a small structural difference in the lobes and inter-lobe linker of these two iron binding proteins [75,76]. Interestingly, lactoferrin seems to have a higher brain uptake than transferrin and OX26 mAb, which may be crucial for the design of more efficient nanocarriers for the brain deliver of drugs [77]. Actually, many works have used nanocarriers functionalized with lactoferrin for cancer, neurodegenerative diseases, and gene therapies [78,79,80,81,82,83,84,85,86,87]. In order to study the real capability of lactoferrin to induce RMT across the brain endothelial cells, Hu et al. used PLA nanoparticles coated with lactoferrin and proved the enhancement of brain uptake compared to nanoparticles alone in intravenously injected mice, using a fluorescent probe (coumarin-6) [78]. With the same purpose, Ye et al. used lactoferrin conjugated with β-cyclodextrins and loaded with a fluorescent dye (IR-775 chloride), and verified the enhancement of brain uptake and bioavailability [79]. The capacity for brain targeting using lactoferrin receptor (LfR) can be also applied, for example, in the treatment of neurodegenerative diseases. With this in mind, Liu et al. developed a nanocarrier system for AD treatment using PCL nanoparticles coated with lactoferrin and loaded with NAP, a neuroprotective peptide [80]. The results showed that, after nasal administration in an AD mice model, PCL-Lf-NAP nanoparticles rescued the balance between acetylcholinesterase and choline acetyltransferase activities and decreased neuronal degeneration in the hippocampus, resulting in memory improvements, as observed in a Morris water maze experiment [80]. Therefore, lactoferrin can be conjugated to nanoparticles, not only to mediate BBB transport, but also for the targeted delivery of the selected molecule/compound to the desired anatomic region in the brain tissue; holding great potential for the treatment of neurodegenerative diseases. Intending to take advantage of these two features at the same time, PLGA-Lactoferrin nanoparticles loaded with urocortin were capable of efficiently reaching the brain and rescuing tyrosine hydroxylase expression in a PD experimental model. As a consequence, it normalized the dopamine content and attenuated the striatum lesion caused by 6-hydroxydopamine in intravenously-injected rats [81]. Considering the potential for gene therapy applications, Somani and colleagues studied 3-diaminobutyric polypropylenimine (DAB) dendrimers functionalized with lactoferrin and loaded with β-galactosidase expression plasmid [82]. After intravenous administration, they observed a significantly higher expression in the brain and lower expression in the other major organs [82]. Transposing this strategy to a concrete application, Huang et al. used polyamidoamine (PAMAM) dendrimers to deliver the human GDNF gene (hGDNF) into a rat model of PD [83]. They observed improved locomotor activity, reduced dopaminergic neuronal cell loss, and enhancement of monoamine neurotransmitter levels [83]. In the cancer field, lactoferrin has also been used to coat nanoparticles and deliver drugs into the brain [84,85,86,87]. As reported in a study, lipid nanoparticles coated with lactoferrin and loaded with tamoxifen were able to pass across the human brain-microvascular endothelial cells (HBMECs) and human astrocytes monolayer co-cultured in transwell devices, which mimic the BBB [84]. Another, very similar, study used PLGA coated with folic acid and lactoferrin loaded with etoposide, a cell cycle inhibitor [85]. In both examples, the developed nanosystems could impair the growth of human primary glioblastoma cells (U87MG) [84,85]. Moreover, Yin et al. synthesized hyaluronic acid nanoparticles functionalized with lactoferrin and loaded with doxorubicin, and studied the effect in intravenously injected C6 glioma-bearing nude mice [86]. They observed an enhanced accumulation in the glioma using real-time fluorescence imaging and, at the same time, an increased survival time of the treated animals [86]. Similar results were obtained in another study with lactoferrin procationic liposomes loaded with doxorubicin in intravenously injected C6 glioma-bearing mice [87]. Collectively, all these works give strong evidence that lactoferrin functionalization is a promising strategy for improving drug accessibility to the brain in a wide range of applications.

#### 2.3.3. Low-Density Lipoprotein Receptors

The primary function of the low-density lipoprotein (LDL) receptor is the removal of highly atherogenic LDL particles from the circulation, being internalized via clathrin-mediated endocytosis [88,89]. Many studies have demonstrated that LDL receptor, LDLR-related protein1 (LRP1), and very low-density lipoprotein receptor (VLDLR) are overexpressed on brain endothelial cells [90]. Therefore, despite also being found in hepatocytes and the sinusoidal endothelium, one can take advantage of these receptors to cross the BBB and deliver drugs inside the brain [90]. Apolipoprotein E (ApoE) is a family member of soluble apolipoproteins [91,92]. This apolipoprotein binds to the LDL receptor and, thus, can be thought as an interesting approach to mediating BBB passage [89]. Several authors have used this strategy to establish new ApoE-coated nanosystems for brain drug delivery [93,94,95,96]. Neves and colleagues functionalized solid lipid nanoparticles with ApoE using two strategies that take advantage of the strong interaction between biotin and avidin [93]. The developed nanosystems were used to protect and transport resveratrol, a bioactive compound with neurological benefits, through the BBB. Brain permeability was evaluated in transwell devices with a hCMEC/D3 cell monolayer, and a 1.8-fold increment in barrier transit was verified for functionalized nanoparticles, when compared with non-functionalized ones [94]. As a proof of concept, Zensi et al. used albumin nanoparticles covalently bound to ApoE and showed that only coated nanoparticles were found in the brain capillary endothelial cells and neurons of intravenously-injected SV 129 mice [95]. Similarly, a study with solid lipid nanoparticles covalently bound to mApoE (residues 141–150 of human apolipoprotein E) in pulmonary-administered mice showed an equivalent brain uptake effect, using a fluorescent dye. They also conducted some permeability studies using a human cerebral microvascular endothelial cell monolayer as an in vitro model, and the results were concordant with what happened in vivo [96].

Another, alternative, method of ApoE targeting is through the coating of nanoparticles with polysorbate 80, because it induces adsorption of ApoE while in systemic circulation [97]. Sun et al. coated PLA nanoparticles with polysorbate 80 and injected intravenously in mice. The results showed that only nanoparticles with the polysorbate 80 coating induced fluorescence in brain tissues [98]. Taking advantage of the targeting efficiency of this nanosystem, Ruan et al. tested this strategy for the design of new drug carriers, using PLA-polysorbate 80 nanoparticles loaded with neurotoxin-1, an analgesic peptide, in intranasal-injected mice [99]. After applying the hot-plate test or the formalin test, which consisted in the analysis of mice reaction to a thermal stimulus or to a localized inflammation induced by formalin, an analgesic effect was successfully achieved [99]. This undoubtedly shows that, upon reaching the brain (although in this study the administration route does not require BBB passage), they were efficient for targeted delivery to the brain cells, suggesting that this strategy ensures BBB transport, as well as coordinated delivery to the desired cell target. Neurodegenerative diseases are also an interesting field of applications to explore this approach [100,101,102]. Wilson et al. developed poly(n-butylcyanoacrylate) nanoparticles coated with polysorbate 80 and loaded with tacrine, a cholinesterase inhibitor used for treating mild to moderate AD. These nanosystems were tested in intravenously-injected rats, and the results showed a higher accumulation of tacrine in the brain compared with other major organs, demonstrating great capacities for AD treatment [100]. The same author also used this nanosystem to encapsulate rivastigmine, a reversible cholinesterase inhibitor used for the treatment of AD, and the results were in agreement with the latter-mentioned study; increasing brain uptake and reinforcing the capacity as a new drug carrier for AD [101]. Furthermore, Jose and coauthors used PLGA nanoparticles loaded with bacoside-A, a therapeutic drug used for the treatment of neurodegenerative diseases, and observed a higher concentration of this drug in the brain of intravenously injected mice [102]. Some authors have also used polysorbates to deliver chemotherapeutic drugs for brain cancer, to increase drug bioavailability, and reduce toxic side-effects [103]. In a work conducted by Wang et al., polybutylcyanoacrylate loaded with gemcitabine could inhibit the growth of C6 glioma cells in vitro, increasing the number of cells in the G0/G1 phase and increasing the survival time of intravenously injected rats [103].

Angiopep-2 can be also used to induce transcytosis of nanoparticles across the BBB, through recognition by LDL receptors. Angiopep-2 is a peptide derived from the kunitz domains of aprotinin and other human proteins, which are ligands for LRP1 and LRP2 [104]. Demeulle and Xin demonstrated that angiopep-2 binds to LRP1, being internalized by brain neuroglial and brain capillary endothelial cells (BCECs) [104,105]. Therefore, this peptide could be a promising and useful molecule to help in brain delivery. In fact, many works have used this strategy to overcome the problem imposed for the BBB in brain drug delivery [106,107,108,109,110,111]. Huile et al. synthesized PCL-angiopep-2 nanoparticles and observed a higher brain uptake compared to unconjugated nanoparticles, which indicates that these nanocarriers were capable of crossing the BBB [106]. Moreover, concerning fungal infections and brain inaccessibility for amphotericin B, Angiopep-2-conjugated polymeric micelles loaded with this drug were developed, to establish a new way of treating brain fungal infections [107]. The results showed that these micelles could enter the BCEC monolayer, and, at the same time, Sprague-Dawley rats showed a superior brain uptake when injected with these nanosystems, compared to micelles without angiopep-2 conjugation [107]. In a subsequent study, the same author tested the therapeutic efficacy of this nanosystem and showed a significant reduction of brain fungal burden and enhanced survival time in an immunosuppressed murine model with intracerebral fungal infection [108]. Coating nanoparticles with angiopep-2 has also been used to increase the concentration in the brain tumor site [109,110,111]. In general, the tumor microenvironment presents acidic pH conditions, due to cancer cell metabolism. With this in mind, Ruan et al. used pH-responsive gold nanoparticles loaded with doxorubicin [109]. The results in glioma-bearing mice showed an increased survival time, indicating that this system was able to pass the BBB and, at the same time, release the drug in the cancer site [109]. A similar effect on the survival time of glioma-bearing mice was obtained using angiopep-2 multi-walled carbon nanotubes loaded with doxorubicin after intravenous administration [110]. Lastly, Huang et al. used this targeting system to increase the efficacy of gene therapy in cancer treatment. In fact, PAMAM dendrimers loaded with tumor necrosis factor-related apoptosis inducing ligand (TRAIL) plasmid were developed, in order to induce apoptosis in brain cancer cells [111]. An enhancement of brain uptake in intravenously injected brain tumor bearing mice was observed, while an extension of the median survival time in twelve days compared to temozolomide administration demonstrated that the developed nanosystem was capable of permeating the BBB, entering the brain cancer cells, and inducing apoptosis [111].

#### 2.3.4. Nicotinic Acetylcholine Receptors

Nicotinic acetylcholine receptors (nAChR) bind the neurotransmitter acetylcholine, playing an important role in the peripheral and central nervous system [112,113,114,115,116,117]. nAChR are widely expressed in the brain in both the pre- and post-synaptic sites of neurons [118,119,120,121]. However, some studies have demonstrated that the expression of these receptors can also be found in the BBB, which could be very promising for designing nanoparticles for brain drug delivery, since they can play a dual role; allowing BBB passage and targeting neuronal cells at the same time [122]. To take advantage of these attractive receptors, RVG29 peptide, a fragment composed of 29 amino acids derived from the Rabies virus glycoprotein, was conjugated on the surface of several nanosystems [123,124,125,126]. This peptide specifically binds to the nAChR expressed by neuronal cells, enabling neurotropic viruses to cross the BBB and infect brain cells [127]. Kim et al. used pluronic-based nanocarriers, conjugated with both chitosan and RVG29 and loaded with β-galactosidase, and increased the enzyme levels inside the brain compared to nanocarriers without RVG29, demonstrating the capacity of this peptide to increase brain uptake [124]. Concerning gene delivery, Liu et al. used PAMAM-PEG-RVG29 dendrimers loaded with a plasmid for luciferase expression [125]. They observed that nanoparticles with RVG29 were able to cross an in vitro model of the BBB composed of BCECs. At the same time, a higher brain uptake and increased expression of luciferase (reporter gene) was observed by luciferase activity assay in intravenously-injected mice, showing the capacity of these nanosystems for gene delivery [125]. These promising developments were used in AD treatment, in a combined therapy using shRNA encoding plasmids and D-peptide [126]. shRNA was used to downregulate an enzyme responsible for the transformation of amyloid precursor protein (APP) in Aβ peptide, while D-peptide was used to inhibit Tau phosphorylation. These two molecules were loaded into dendrigraft poly-L-lysines (DGLs) functionalized with RVG29 and showed a reduction of neurofibrillary tangles, leading to a memory loss rescue in intravenously injected APP/PS1 transgenic mice [126].

Another valid strategy for brain delivery is the use of snake neurotoxin candoxin (CDX) peptide. This fragment is composed by 16 amino acids from the loop II region of snake neurotoxin candoxin and binds to nAChR with high affinity [128,129]. Some authors adopted this strategy to deliver drugs inside brain [130,131]. Concerning brain glioma, Wei et al. synthesized liposomes modified with CDX and c(RGDyK), which is a ligand for integrins highly expressed in the tumor cells, and the results showed the ability of this nanosystem to cross a BCEC monolayer and, at the same, to increase the median survival time in nude mice bearing glioma [130]. Furthermore, in another study, co-delivery of TRAIL encoding plasmid and paclitaxel using two separate nanosystems was tested [131]. Paclitaxel was loaded in PLA nanoparticles functionalized with CDX, and TRAIL encoding plasmid was loaded into polyethylenimine modified with RGD [131]. The results demonstrated the synergistic effect of these two molecules, observed by an increase in the median survival time of an intravenously-injected glioblastoma-bearing mice model [131].

#### 2.3.5. αvβ3 Integrin Receptors

Integrins are heterodimeric receptors of two transmembrane subunits (α and β), which are assembled in a noncovalently manner, forming an extracellular globular head, and with the remaining portions forming two rod-shaped tails that spin the plasma membrane [132,133,134,135,136]. These receptors are involved in many crucial cellular processes, such as adhesion, migration, differentiation, survival, assembly of the extracellular matrix, growth factor signaling, organization of the cytoskeleton, and cytoskeleton-mediated processes, such as contraction, endocytosis, and phagocytosis [137]. However, they have an important role in the development of many diseases, such as neoplasia, tumor metastasis, and immune dysfunction [138,139]. In particular, αvβ3 integrin has been associated with angiogenesis in malignant gliomas via basic fibroblast growth factor (bFGF) and tumor necrosis factor α (TNF-α) [57,58]. Therefore, this receptor is very useful for targeting brain tumor cells. RGD peptide is composed of arginine-glycine-aspartic acid and has the capacity for binding to αvβ3 integrin. Hence, RGD can be used to coat nanoparticles, to achieve the desired brain targeting [140]. There are some studies in the literature implementing this approach for cancer therapy [141,142]. Nevertheless, this system was not used solely for cancer therapy, but was also implemented for imaging, to generate a better contrast and, consequently, for the earlier detection of certain diseases that are highly dependent on the timing of the diagnosis for the success of the treatment. In this context, Richard and coauthors synthesized iron oxide nanoparticles decorated with RGD peptide for brain tumor imaging. The results showed an accumulation in the cancer site and a greater contrast, allowing a better detection of the tumor in the brain of a xenografted U87MG mouse tumor model [141]. In an attempt at conjugating diagnosis with therapy, Sonali et al. developed a proof-of-concept study using theranostic liposomes decorated with RGD peptide, for the delivery of docetaxel combined with quantum dots, in intravenously-injected mice. The results showed an enhancement of brain uptake and imaging contrast compared to liposomes alone [142].

#### 2.3.6. CD13/APN Receptor

CD13/APN is a membrane-bound protein homodimer responsible for several biological functions [143]. This receptor belongs to the Zn-binding metalloproteinase superfamily, having a crucial role in angiogenesis and tumor cell migration [143]. The increased activity of this protein in cancer patients when compared with healthy individuals is associated with tumor progression and growth [59]. Thus, NGR peptide, which is composed of asparagine-glycine-arginine amino acids, has emerged as a promising target molecule for this CD13/APN receptor found in the tumoral microenvironment, because it binds specifically to cancer cells and not to other CD13 rich tissues [144]. Due to its specificity for cancer sites, some authors have used this strategy in the development of drug carriers for brain cancer delivery [145,146,147,148]. Kang et al. developed docetaxel-loaded PLGA nanoparticles for brain delivery in a mice model bearing intracranial U87 glioma [145]. This nanosystem was capable of reaching the glioma site in higher quantities, showing anti-angiogenesis activity, and a significant extension of the mice survival time [145]. Pastorino et al. tested aGD2-SIL(DXR) and NGR-SL(DXR) liposomes loaded with doxorubicin [146]. The results showed a decrease in tumor angiogenesis and an increase in the median survival time of intravenously-injected neuroblastoma mice, demonstrating a synergistic effect of these two types of nanosystem [146]. NGR receptor has been also used to study its potential for gene therapy applications. As such, An and colleagues developed nanospheres loaded with siRNA for luciferase [147]. They modified nanoparticles with NGR peptide, and this system was sensible to glutathione activity, which is highly expressed in tumor cells [147]. The results showed that nanoparticles reached the brain of a mice-glioma model and were able to downregulate the luciferase expression, indicating excellent capacities for gene therapy [147]. As previously mentioned, it is also necessary to make various improvements in the diagnosis imaging field, to create better contrast between the surrounding tissue and the site of interest. In this context, Huang et al. used quantum dots modified with NGR to target tumor cells and their vasculature, taking advantage of CD13 expression [148]. The enhancement of the fluorescence observed in the tumor cells and vasculature of a intravenously-injected rat glioma model compared to quantum dots alone, indicated that NGR target cancer sites and could be used as an important adjuvant for brain cancer imaging [148].

## 3. Marketed Formulations and Clinical Trials

In recent years, several nano-based drug delivery systems have been approved by the Food and Drug Administration (FDA) for the prevention and treatment of a wide spectrum of illnesses, including cancer and infectious diseases. The number of patents and marketed formulations in the nanomedicine field is significantly increasing, and many clinical trials are currently taking place. Until now, no one formulation based on nanoparticles has received approval for clinical use in the prevention and treatment of CNS diseases, although there are some clinical trials that are currently ongoing, and others are planned [149,150]. Thus, an ongoing phase-I trial (NCT03603379) of anti-EGFR-immunoliposomes loaded with doxorubicin will compare the ratio between the concentration of doxorubicin in the cerebro-spinal fluid and in the peripheral blood after intravenous administration of the formulations [151]. One single-center open label pilot (NCT03815916) is currently ongoing and is testing the efficacy of gold nanocrystals in Parkinson’s disease [152]. Moreover, two predicted clinical trials are expected to occur in the future. The clinical trial NCT03806478 is a phase-II trial that will evaluate the efficacy of intranasal delivery of APH-1105 for the treatment of mild to moderate Alzheimer’s in adults [153]. In the phase-II clinical trial, NCT03843710, the efficacy of gold nanocrystals will be evaluated in amyotrophic-lateral sclerosis [154]. If the results of the clinical trials mentioned above are in accordance with the varied panoply of studies mentioned in this review in terms of brain delivery efficacy, this might help in the translation of nanotechnology “from the bench to the clinic”. Furthermore, this could generate a positive feedback loop, whereby new clinical trials can be designed and launched. In that sense, these pioneer studies are crucial for the future development of nano-approaches for the treatment of neurological diseases.

## 4. Conclusions

In conclusion, intensive research has been devoted to find promising and attractive brain drug delivery strategies. The necessity of circumventing the physical barrier that the BBB represents to the delivery of drugs into the brain in high concentrations is undoubtedly a great challenge in improving current therapies. Here, we have reviewed exciting progress and research advances within the context of the brain delivery of pharmaceutical bioactive compounds, as well as imaging contrast agents, conjugated in nanotheranostic applications. Active targeting has been summarized as being particularly important when designing solutions to achieve this goal, since this strategy allows directing the nanoparticles to the desired site of action, by modifying the surface of NPs with molecules that may be recognized specifically by receptors or transporters overexpressed in the brain, such as transferrin, lactoferrin, LDL, nAChR, and αvβ3 integrin receptors, or glucose, glutathione, and amino acids transporters. Despite the enormous efforts in the nanotechnology field for improving current diagnostic and therapeutic strategies for the treatment of neurological disorders, nanomedicine has yet to make its mark in clinical studies. In this context, we believe that the clinical translation of this pre-clinical evidence and findings should be fully exploited in the near future, in order to promote the introduction of nano-based formulations into the market. As a result, these promising and novel solutions, based on site-specific brain drug-delivery, may improve the current therapy for CNS disorders.

## Figures and Tables

**Table 1:** Types of actively targeted nanoparticles used for brain delivery.

Targeting Effector/Ligand	NPs Type	Composition	Therapeutic Agent	Size (nm)	Zeta Potential (mV)	EE (%)	Administration Route	In Vitro/In Vivo Results	Refs
Adsortive-mediated transcytosis									
Lectin	Polymeric nanoparticles	PLA/PEG/WGA; PLA/PEG/STL; PLGA/PEG/STL; PLGA/PEG/OL	VIP, bFGF, HLP, UCN	85 to 130	−30 to −15	70 to 75	Intranasal	- increased brain uptake in rats compared to NPs with no lectin; - improved spatial learning and memory in AD rats; - enhanced neuroprotection in schizophrenic or hemiparkinsonian rats.	[3,4,5,6,7]
Cardiolipin	Liposomes	SM/Chol/CL	n.i.	100 to 150	−38 to +50	n.i.	intraperitoneal	- reduced cell viability and Tau phosphorilation in mouse neuroblastoma cell lines; - reduction of Aβ levels and Tau phosphorilation in plasma and brain of APP/PS1 transgenic mice; - high affinity for Aβ fibrils association.	[8,9,10,11]
Heparin	Magnetic nanoparticles	Fe_3_O_4_/Heparin	n.i.	70	−50	n.i.	n.i.	- high affinity for Aβ fibrils association and protection of neuronal cells against Aβ toxicity.	[12]
CPPs	Polymeric nanoparticles; Polymeric micelles; Quantum dots	PLA/PEG/Penetratin; MPEG/PCL/TAT; ZnS:Mn/ZnS/TAT	siRNA for Raf-1/CPT	60 to 200	−5 to +15	n.i.	intravenous, intranasal, intra-arterial	- enhanced brain uptake in SD rats; - inhibition of tumor growth in rat model of malignant glioma; - specific brain delivery for QDs theranostic.	[13,14,15]
Transporter-mediated transcytosis									
Mannose	Lipid nanoparticles; Cationic HSA nanoparticles	GMS/SA/SL/MAN; HSA/EDA/Mannose	DT, DOX	90 to 100	−15 to −10	75 to 85	intravenous	- increase of DT brain uptake in mices; - higher DOX transport across bEnd.3 monolayer and uptake by U87MG glioblastoma cells; - reduction of tumor size of glioma-bearing mice.	[16,17]
Glutathione	Liposomes; Polymeric nanoparticles	EPC/CHOL/GSH; PLGA/GSH; PEI/GSH	RBV, PTX	80 to 300	−40 to −5	45	intravenous, intranasal	- increase of RBV and PTX brain uptake in rats; - receptor-mediated uptake in human, bovine, and porcine cerebral microvascular endothelial cells; - enhanced passage across the hCMEC/D3 cells.	[18,19,20]
Amino acids	Liposomes; Lipid nanoparticles	SL/Chol/TPGS/GLU; DSPE/PEG/ PHE	DOX	80 to 165	−35 to −15	75	intravenous	- higher uptake by C6 glioma cells and accumulation of DOX in brain of mice.	[21,22]
Receptor-mediated transcytosis									
Transferrin	Lipid nanoparticles; Polymeric nanoparticles; Carbon dots; Albumin nanoparticles; Liposomes; Silica/polymeric/magnetic; Dendrimers	Compritol/SA/Tf; PLGA/Tf; Carbon powder/Tf; HSA/Tf; DSPE/PEG/Tf; PLGA/TMOS/Fe_3_O_4_/Tf; DAB/Tf	SQV, LOP, α-M, DOX/PTX, pβ-Gal	10 to 200	−30 to −5	20 to 90	intravenous	- Higher uptake and transcytosis across human brain-microvascular endothelial cell monolayers; - C-dots with Tf can enter easily in the CNS of injected zebrafish; - Higher LOP brain uptake and anti-nociceptive effects in the tail-flick test in ICR (CD-1) mice; - Tf-liposomes improved brain delivery of α-M in rats; - Strong anti-glioma activity of DOX/PTX Tf-NPs in mice; - higher β-Gal expression in the brain after Tf-dendriplex administration in mice.	[23,24,25,26,27,28,29]
OX26 mAb	Albumin nanoparticles; Lipid nanocapsules; Polymeric nanoparticles; Liposomes	HSA/OX26; SL/PC/PE/OX26; PLGA/ DE2B4/OX26; POPC/PEG/OX26; DSPC/Chol/DSPE-PEG/19B8Mab/OX26	LOP, iAβ_5_, pTH	115 to 170	−35 to −3	60	intravenous	- higher LOP brain uptake and anti-nociceptive effects in the tail-flick test in ICR (CD-1) mice; - increased brain uptake compared to non-targeted nanocapsules in rats; - increased uptake of iAβ_5_ in porcine brain capillary endothelial cells; - pTH-NPs enter CNS, rescue gene expression and revert motor impairment in PD rats.	[26,30,31,32,33]
Lactoferrin	Polymeric nanoparticles; Cyclodextrins; Dendrimers; Lipid nanoparticles; Liposomes	PLGA/PEG/Lf; PCL/PEG/Lf; PLGA/PEG/Lf; PLGA/FA/Lf; HA/Lf; β-CDs/PEG/Lf; DAB/Lf; PAMAM/PEG/Lf; BA/TP/Cac/TMX/Lf; EPC/CHOL/CHETA/Lf	UCN, ETP, NAP, DOX, pβ-Gal, GDNF, BCNU, DOX	90 to 210	−30 to +40	35 to 98	intravenous, intranasal,	- enhanced brain uptake and bioavailability in mice; - neuroprotection and memory improvements in AD mice model after NAP-NPs administration; - attenuation of striatum lesion caused by 6-OHDA in PD rats after UCN-NPs administration; - improved locomotor activity and reduced neuronal cell loss in PD rats by GDNF-dendrimers; - higher β-Gal expression in the brain after Lf-dendrimers administration in mice; - enhanced permeation across BBB cell monolayer and inhibition of U87MG glioblastoma cell growth; - higher accumulation in tumor cells and extended survival time of glioma-bearing rats by DOX-NPs.	[34,35,36,37,38,39,40,41,42,43]
ApoE	Lipid nanoparticles; Albumin nanoparticles	CP/DSPE-PEG/ApoE; HSA/PEG/ApoE; SL/PC/mApoE	RSV	120 to 250	−55 to −15	85 to 98	intravenous, intrapulmonary	- higher permeability of RSV-NPs through hCMEC/D3 monolayers; - only ApoE-NPs found in brain capillary endothelial cells and neurons of SV 129 mice; - enhanced brain target via pulmonary administration in mice.	[44,45,46]
Polysorbate 80	Polymeric nanoparticles	PLA/PS80; PBCA/PS80; PLGA/PS80	NTX-1, TC, RVT, BA, GEM	35 to 210	−40 to −10	50 to 60	intravenous, intranasal	- only PS80-coated NPs found in brain tissues of mice; - antinociceptive effects after NTX-1-loaded NPs administration in mice; - higher accumulation of TC-NPs, RVT-NPs and BA-NPs than free forms in brain of rats; - GEM-NPs inhibit C6 glioma cells growth and increase survival time of rats.	[47,48,49,50,51,52]
Angiopep-2	Polymeric nanoparticles; Micelles; Gold nanoparticles; Carbon nanotubes; Dendrimers	PCL/PEG/AP-2; PE/PEG/AP-2; Au/PEG/AP-2; MWNTs/PEG/AP-2; PAMAM/PEG/AP2	AMB, DOX, TRAIL	10 to 200	−20 to +15	80	intravenous	- higher accumulation of AP-2 NPs in the brain of SD rats, compared to non-functionalized ones; - enhanced permeation of AMB across BBB and brain uptake in SD rats for brain fungal burden; - increased survival time of glioma-bearing mice after DOX-loaded Au NPs and MWNTs; - enhanced brain uptake and survival time of tumor-bearing mices after TRAIL-dendrimers.	[53,54,55,56,57]
RVG29	Polymeric nanoparticles; Dendrimers;	PLGA/RVG29; PAMAM/PEG/RVG29; DGL/PEG/RVG29; Pluronic/PEG/RVG29	CPT, pLuc, shRNAs, β-Gal,	110 to 150	−3 to 12	40 to 80	intravenous	- enhanced apparent brain delivery of NPs in the presence of RVG29 peptide; - increased expression of LUC gene in mice brain; - reduction of neurofibrillary tangles and rescue of memory loss in AD transgenic mice; - β-Gal delivered and accumulated efficiently in mice brain.	[58,59,60,61]
CDX	Liposomes; Polymeric nanoparticles	HSPC/DSPE-PEG/CDX; PLA/PEG/CDX	DOX, PTX	95	n.i	95	intravenous	- ability for crossing the BBB monolayer and enhanced median survival time in glioma-bearing mice after DOX and PTX-loaded NPs-CDX administration.	[62,63]
RGD	Magnetic nanoparticles; Liposomes	Fe_2_O_3_/PEG/RGD; DPPC/Chol/TPGS/RGD	DT/QDs	40 to 180	−20 to +1	70	intravenous	- accumulation in the cancer site, generating a MRI contrast for mice brain tumor imaging; - enhanced brain uptake and imaging after DT/QDs-loaded liposomes administration in mice.	[64,65]
NGR	Polymeric nanoparticles; Liposomes; Quantum dots	PLGA/PEG/NGR; HSPC/CHOL/DSPE-PEG/NGR; OEI/PEG/NGR; CdSe/ZnS/PEG/NGR	DT, DOX, siRNA-Luc	10 to 130	15	50	intravenous	- anti-angiogenesis and prolonged survival time in mice bearing intracranial glioma by DT or DOX-NPs; - downregulation of reporter gene (Luciferase) in glioma cells in mice; - enhanced brain cancer imaging in rat glioma model after QDs-NGR administration.	[66,67,68,69]

List of abbreviations: *β*-*gal*: *β*-*galactosidase*; Apo-E: *Apolipoprotein E*; AD: Alzheimer’s; BBB: blood–brain barrier; Chol: cholesterol; DOX: doxorubicin; DSPE: 1, 2-Distearoyl-sn-glycero-3-phosphoethanolamine-Poly(ethylene glycol); FA: folic acid; NPs: nanoparticles; %EE: %encapsulation efficiency; MRI: magnetic resonance imaging; PAMAM: Poly(amidoamine); PTX: paclitaxel; PC: phosphatidylcholine; PLGA: poly(lactic-co-glycolic acid); PEG: polyethylene glycol; QD: quantum dots; siRNA: small interfering RNA; SD: *Sprague-Dawley.*
